# Manifestation of tuberculosis in patients with human immunodeficiency virus: A large Indian study

**DOI:** 10.4103/1817-1737.32231

**Published:** 2007

**Authors:** S. Rajasekaran, A. Mahilmaran, S. Annadurai, S. Kumar, K. Raja

**Affiliations:** Govt. Hospital of Thoracic Medicine, Tambaram Sanatorium, Chennai, India

**Keywords:** Clinical manifestations, human immunodeficiency virus, tuberculosis

## Abstract

**BACKGROUND:**

Government Hospital of Thoracic Medicine, Tambaram Sanatorium, Chennai, is the largest HIV-care center in South East Asia. As many as 29,300 HIV patients visited this center at least once in the year 2005 for care and support.

**OBJECTIVES:**

Clinical manifestations and the modes of presentation of tuberculosis were assessed among 12,750 adult and adolescent patients with human immunodeficiency virus (HIV) attending the hospital for the first time.

**MATERIALS AND METHODS:**

Database of Hospital Information System, specially evolved for managing patients afflicted with tuberculosis and HIV, was utilized. The particulars confined to patients with tuberculosis and HIV co-infection who visited the hospital for the first time from January to December 2005 were considered for the analysis. Proportion test and Chi-square test with Yates correction were done.

**RESULTS:**

As many as 12,750 adult and adolescent HIV-confirmed patients were screened for the possible presence of tuberculosis. Out of them, 4,383 (34.4%) patients had tuberculosis. Among them, 2,448 (55.9%) had pulmonary tuberculosis, and the remaining 1,935 (44.1%) had either disseminated or extra-pulmonary tuberculosis (P<0.001). Positive sputum-smear microscopy for acid fast bacilli was evident in 1,363 (31.1%) patients; however, it was significantly lower compared to positive smear rate of 44% in HIV patients (P< 0.001).

**CONCLUSION:**

Tuberculosis was found to be the predominant co-infection among the symptomatic patients infected with HIV attending the largest care center for the first time in India. Advanced tuberculosis, disseminated tuberculosis and sputum smear negative pulmonary tuberculosis were the presenting clinical manifestations in 44% of the patients, as they had moderate to advanced immunosuppression. Early detection of tuberculosis co-infection is absolutely necessary.

The human immunodeficiency virus (HIV) epidemic in India and other resource-limited countries is posing greater challenges to the containment of tuberculosis in HIV-afflicted individuals and collectively to the very control of tuberculosis. In the presence of infection with HIV, tuberculosis manifests in many ways; there may be primary tuberculosis, reactivated tuberculosis, or some patients may suffer from new TB infection (reinfection).[1] Studies conducted in rural[2] and urban[3–6] India revealed a rising trend of HIV-TB co-infection. This is likely to have negative impact on the well-functioning TB-control program and the existing AIDS-control program. Clinical features of HIV-associated pulmonary tuberculosis in adults are frequently atypical, particularly in the late stage of HIV infection, with noncavitary disease, lower lobe infiltrates, hilar lymphadenopathy and pleural effusion.[7,8] African countries affected by both TB and HIV are experiencing a disproportionate increase in smear-negative tuberculosis[9] and extra-pulmonary tuberculosis.[10] Diagnostic algorithms and treatment protocols must be developed for each country, taking into consideration various factors, including the commonly occurring opportunistic infections. This study provides an insight into the prevalence and clinical manifestations of HIV-TB co-infection among the patients attending for the first time the largest health care setting in India that provides care and support to such patients.

## Materials and Methods

Govt. Hospital of Thoracic Medicine, Tambaram Sanatorium, Chennai (GHTM, Tambaram), is the largest voluntary counseling and testing center in the country, providing HIV counseling and testing to more than 2,400 patients a month. All these patients are also screened for the possible coexistence of tuberculosis by performing sputum-smear microscopy for acid fast bacilli (AFB) and radiological investigations including chest radiography. Other specimens given by patients from extra-pulmonary sites are also subjected to smear microscopy.

Opportunistic infections were identified predominantly by laboratory investigations. Clinico-radiological methods and oxygen saturation were utilized in detecting pneumocystis carinii pneumonia. Computerized tomography and magnetic resonance imaging scans of brain helped in sorting out many central nervous system manifestations. Fine needle aspiration cytology and histopathological examination of the biopsied specimens were also resorted to wherever necessary.

Computerized database of Hospital Information System provides patient records and all the data analysis of various aspects of HIV-TB co-infection. This study is confined to the evaluation of manifestations of TB in adult and adolescent HIV patients who attended GHTM for the first time during 2005. Proportion test was done with the null hypothesis value of 50% to compare pulmonary *vs.* extra-pulmonary TB and the sex ratios. Chi-square test was done to compare the distribution among various age groups and smear-positive rates.

## Results

Twenty-nine thousand three hundred and eighty-six patients with HIV disease attended GHTM, Tambaram, at least once in 2005. As many as 13,348 patients visited the institution for the first time; out of them, 12,750 were aged 15 years and above, and they formed the study population for further analysis.

Among all the opportunistic infections that coexisted with 12,750 HIV patients, oral candidiasis (52%) and *Pneumocystis carinii* (jiroveci) pneumonia (42%) were found to be more frequent than tuberculosis (34%). Lower respiratory tract infection, including pneumonia, was found in 22% of the patients [Table 1].

**Table 1:** Opportunistic infections

Common ois	Patients	%
Oral candidiasis	6590	52
PCP	5401	42
Tuberculosis	4383	34
LRI/pneumonia	2761	22
Oesophageal candidiasis	1337	10
Diarrhoea / pathogens	1033	8
Scabies	611	5
Hepatitis	313	2
Herpes zoster / simplex	222	2
Malaria	120	1
Cryptococcois	65	<1

Among 4,383 HIV-TB patients, 74.5% were males and the rest (25.5%) were females [Table 2] and the difference was statistically significant (*P*<0.001). As many as 86.5% of HIV-TB patients were in the 15-44 age group, which was significantly higher (*P*<0.001) as compared to other age groups. However, among females, 40% of HIV-TB patients were in the 15-29 age group, which was two times significantly higher as compared to males (20%) (*P*<0.001).

**Table 2:** Age and sex distribution of human immunodeficiency virus patients with tuberculosis

Age group	Men	Women	Total	
			
			Patients	%
15–29	646	447	1093	24.9
30–44	2149	550	2699	61.6
45–59	431	112	543	12.4
>59	40	8	48	1.1
Total	3266	1117	4383	100
	74.5%	25.5%		

As many as 2,448 (56%) patients with HIV had pulmonary TB, which was statistically significantly higher as compared to 1,935 (44%) patients detected to have extra-pulmonary TB and disseminated TB [Table 3] (*P*<0.001). However, it is also true that unlike in non-HIV tuberculosis patients, almost every second patient was found to suffer from either extra-pulmonary TB or disseminated TB, apart from his/her HIV disease.

**Table 3:** Types of tuberculosis

Types of tuberculosis	Patients	%
Pulmonary tuberculosis	2448	56
Pulmonary tuberculosis (PTB) and extra PTB	620	14
Extra pulmonary tuberculosis	1315	30
Total	4383	100

Lymph nodal tuberculosis and pleural tuberculosis were found to dominate the extra-pulmonary manifestations [Table 4]. Intrathoracic lymph nodes, hilar and mediastinal, were detected in 1,523 (79%) patients.

**Table 4:** Manifestations of extra-pulmonary tuberculosis

Extra pulmonary tuberculosis	Patients	%
Intra thoracic lymphnodes	1523	79
Extra thoracic lymphnodes	583	30
Pleural disease	1063	55
Pericardial effusion	113	6
Tuberculosis abdomen	48	3
Tuberculosis meningitis	30	2
Tuberculoma	19	1
Others	97	5

Total extra pulmonary TB: 1935

Sputum smear microscopy for AFB was performed in all the patients. Of the 3,068 HIV patients, 1,383 (44%) were found to have smear-positive pulmonary tuberculosis [Table 5]. Sputum smear positive yield was significantly lower (31%) when all 4,383 HIV-TB patients were considered as compared to 44% of HIV and TB patients (*P*<0.001).

**Table 5:** Sputum microscopy for acid fast bacilli

Sputum acid	All types of TB		Pulmonary TB	
		
fast bacilli	patients with HIV		with HIV	
	Patients	%	Patients	%
Positive	1363	31	1363	44
Negative	3020	69	1705	56
Total	4383	100	3068	100

TB - Tuberculosis, HIV - Human immunodeficiency virus

## Discussion

The fight against tuberculosis would always remain incomplete without addressing the issues related to the control of HIV/AIDS. This study clearly brings out the important message that at least 34% of the HIV patients were found to have tuberculosis co-infection at the time of detecting or confirming their HIV disease. Significantly, a lower number of women living with HIV were found to attend the hospital, reflecting their current health remedy seeking behavior. Further, they need the support of their family members to travel long distances to attend the health institutions providing care and support to people living with HIV.

This study, apart from confirming the rising trend of HIV-TB co-infection in Tamil Nadu and other states of India where the prevalence of HIV is high, also confirmed the trend of shifting of youth peak prevalence of TB in HIV patients to the lower age group. Unlike the non-HIV immunocompetent patients, tuberculosis was found to occur more commonly in young and middle-aged adults.

Other vital factors that came out of this study were the type and severity of tuberculosis detected among the HIV patients attending GHTM, Tambaram, for the first time. While the usual pulmonary tuberculosis was detected only to the extent of 56% of HIV-TB study population, disseminated TB and extra-pulmonary TB were witnessed in 14% and 30% of the patients respectively. Increasing frequency of dissemination of tuberculosis was observed with extra-pulmonary manifestations in several developing countries[7–14] as the hallmark of advanced HIV disease. This is the resultant of unrecognized[15] and demonstrable[16] Mycobacteremia in severely immunosuppressed patients.

Among all the HIV-TB patients, sputum smear positive detection rate was low (31% only). Advanced HIV disease is often associated with sputum smear negative pulmonary tubercuosis,[1,7,9] atypical radiographic pictures[17–19] and extra-pulmonary spread. Unrecognized tuberculosis in patients with HIV disease has far-reaching consequences, including delayed diagnosis, unacceptable therapeutic delay[20] and even rapid progression to ‘untreatable TB.’

## Conclusion

Tuberculosis was found to be the predominant co-infection (34%) among the symptomatic HIV patients attending the largest HIV care center in India for the first time. Significantly, 44% patients were reporting with clinical manifestations of advanced tuberculosis, indicating the associated moderate to severe immunosuppression. Smear-negative pulmonary tuberculosis and extra-pulmonary tuberculosis are likely to pose diagnostic dilemma to clinicians used to treat tuberculosis in non-HIV patients. Early detection of varied forms of tuberculosis among HIV seropositives is absolutely necessary for instituting appropriate antituberculosis treatment well before the disease gets disseminated.
